# A minimally invasive lab protocol for fibroblasts isolation from 1-mm skin punch biopsies in pediatric patients

**DOI:** 10.1371/journal.pone.0350828

**Published:** 2026-06-22

**Authors:** María Heredia-Torrejón, Begoña Puga-López, María Dolores Guerrero-López, Raúl Montañez, Alfonso María Lechuga-Sancho

**Affiliations:** 1 Department of Child and Mother Health and Radiology, Instituto de Investigación e Innovación Biomédica de Cádiz (INiBICA), Medical School, Universidad de Cádiz, Cádiz, Spain; 2 Research Unit, Instituto de Investigación e Innovación Biomédica de Cádiz (INiBICA), Hospital Universitario Puerta del Mar, Universidad de Cádiz, Cádiz, Spain; 3 Department of Molecular Biology and Biochemistry, University of Málaga, Andalucía Tech, Málaga, Spain; 4 Division of Endocrinology, Department of Pediatrics, Hospital Universitario Puerta del Mar, Universidad de Cádiz, Instituto de Investigación e Innovación Biomédica de Cádiz (INiBICA), Cádiz, Spain; University of Kentucky College of Medicine, UNITED STATES OF AMERICA

## Abstract

Skin biopsies provide a valuable source of culturable cells for a broad range of clinical and biomedical research applications. However, conventional methods for establishing primary cell cultures typically require relatively large tissue specimens obtained through invasive procedures that involve intradermal anesthesia, suturing, and subsequent wound care. These factors are of particular concern when working with pediatric populations. To address these limitations, we developed a simplified protocol that reduces patient discomfort by minimizing the punch biopsy diameter to as little as 1 mm. Despite the markedly smaller sample size, this method consistently enables the successful establishment of primary fibroblast cultures, with no complications reported to date. This streamlined approach is compatible with basic cell culture facilities and provides a reliable source of fibroblasts for downstream applications. Overall, this protocol represents a meaningful advancement in both pediatric research and clinical practice.

## 1. Introduction

Patient-derived cell cultures constitute experimental models that faithfully represent human biology. They are indispensable for elucidating physiological processes, their disruption in disease and for establishing robust molecular diagnostics. However, obtaining suitable patient-derived primary cells remains a major practical challenge in biomedical research, as not all human cell sources are equally accessible or physiologically representative. While peripheral blood mononuclear cells (PBMCs) are easily procured, providing a common source of germline DNA for genetic testing or RNA, proteins and metabolites for omic studies, they are generally unsuitable for long-term culture, precluding functional assays. Moreover, their utility is limited when a non-hematopoietic genomic or enzymatic profile is essential. For instance, primary culturable cells are mandatory for the definitive diagnosis of certain inborn errors of metabolism, the accurate detection of low-level somatic mosaicism, and for the establishment of an uncompromised cell lineage in patients with hematological malignancies or in those who have undergone allogeneic or stem cell transplantation, where acquired somatic mutations can entirely confound germline analysis [1]. Furthermore, many biomedical applications require an uncompromised lineage of patient-derived cells with long-term in vitro expansion potential rather than readily available samples. For instance, the functional validation of genetic variants within the patient’s native genetic background depends on the availability of such cells. Therefore, in these scenarios, human dermal fibroblasts (HDFs) derived from skin biopsies emerge as the preferred robust primary cell model, offering the necessary genetic stability and cultivability for high-fidelity studies.

Alternative cell models, although more convenient, present important limitations. While immortalized cell lines offer unlimited division and ease of maintenance, their utility in translational studies is severely restricted by accumulated genetic drift and alterations inherent to the immortalization process, which often misrepresent native physiological processes [2]. Similarly, induced pluripotent stem cell (iPSC)-based approaches, although capable of generating disease-relevant cell types [3], remain technically complex, time-consuming, and prone to incomplete differentiation and the retention of epigenetic memory [4,5]. Primary cells, conversely, are sourced directly from the patient, preserving both their native genetic background and crucial epigenetic signals, thereby providing a highly accurate representation of in vivo disease conditions, despite replicative senescence [6]. Consequently, obtaining culturable primary cells via skin biopsy is still a reference standard for high-fidelity biomedical research.

Within this framework, HDFs obtained from skin biopsies stand out as a robust and versatile primary model. They are mesenchymal cells derived from the embryonic mesoderm that reside within the dermal layer of the skin. In culture, HDFs exhibit a characteristic spindle-shaped morphology and express canonical stromal protein markers, including Vimentin (a general mesenchymal intermediate filament), Fibronectin, and TH-1 (CD90) [7]. Recent advances in single-cell transcriptomics have revealed the functional and transcriptional heterogeneity of fibroblasts [7,8], with distinct subtypes exhibiting different marker expression profiles and specialized roles in tissue homeostasis, wound healing, fibrosis, and aging [9–13]. Compared to peripheral blood, where transcriptional activity is dominated by a limited subset of highly expressed genes, fibroblasts exhibit a far more complex transcriptional landscape [14]. This transcriptomic diversity renders fibroblasts particularly valuable for biomarkers discovery and for the functional characterization of gene variants, as they express a broader and more physiologically relevant repertoire of transcripts than blood-derived cells. Nonetheless, as gene expression is tissue-specific, it is essential to verify that the gene of interest is actually expressed in the skin before performing a biopsy; this can be readily assessed using public databases such as Genotype-Tissue Expression (GTEx) Project [15] or UniProt [16] to ensure the biological relevance of the fibroblast model for the specific study.

Despite the acknowledged value and extended use of primary HDFs, conventional skin biopsy protocols (typically using 3–4 mm punches) raise ethical and practical concerns in research settings. Standard procedures include intradermal anesthesia, post-procedural suturing and wound care. The inherent discomfort, coupled with the potential lack of outcome guarantee, results in an unfavorable risk-to-benefit ratio, particularly in pediatric cohorts [17]. Furthermore, skin biopsies are generally reserved for definitive histopathological evaluation, a requirement for which these larger specimens are necessary. However, the use of large specimens solely for cell culture often leads Institutional Review Boards (IRBs) to question the justification for such an invasive sampling method when no histopathology is intended. This constraint limits the widespread use of HDFs in research. To overcome this critical limitation, minimally invasive 1-mm punch biopsies have recently emerged, primarily for direct molecular or omic analysis [18,19]. Although unsuitable for histopathological evaluation, we demonstrate that these reduced-biopsies provide sufficient material for the successful establishment and expansion of primary HDFs cultures. Thereby, the use of these reduced-biopsies, as a standard for HDFs culture, may broaden the potential scope of diagnostic and research applications of primary HDFs cultures, making the acquisition of high-fidelity cellular collections ethically and practically feasible.

Once the tissue explant (TE) has been obtained, the next critical step involves its processing to cultivate fibroblasts. For this purpose, two well-established strategies are commonly employed: *enzymatic dissociation* and *direct explant culture*. The enzymatic approach typically entails gentle overnight digestion with dispase to separate the epidermis and dermis, followed by approximately one hour of collagenase treatment to degrade the extracellular matrix and release fibroblasts [20]. This method enables the rapid recovery of primary HDFs; however, it is technically demanding, as deviations in enzyme concentration or incubation time may reduce cell viability. Furthermore, the multiple handling steps involved increase the risks of contamination and sample loss. Alternatively, the direct explant culture technique relies on mechanically fragmenting the TE into small pieces and allowing them to adhere to the culture plate through partial desiccation. Fibroblasts then migrate and proliferate from the tissue fragments. This direct explant culture is considerably simpler and less susceptible to contamination. Its main limitations are the longer initial times and the potential failure of the tissue fragments to adhere properly. The latter may prevent the establishment of the culture [21]. However, the successful performance of this approach results in a TE releasing additional fibroblasts upon successive passages.

Building on these approaches, we developed and validated a protocol for the isolation of HDFs from 1-mm TE. This technique not only minimizes invasiveness and patient discomfort, but also aligns experimental procedures with stringent ethical standards, without compromising the quality or reproducibility of the resulting primary cell cultures. To promote its adoption in both clinical and research environments, we further streamlined the direct explant culture method, reducing procedural complexity and failure risks. In conclusion, these refinements have demonstrated 98% culture success rates, strengthening the applicability of primary HDFs models for translational and personalized medicine research. Furthermore, it has already been used in an experimental setting successfully [22].

## 2. Materials and methods

The protocol described in this peer-reviewed article has been published on protocols.io [23], and is included for printing as S1 File with this article. As stated above, it has also been used in research published in a peer-reviewed journal [22]. A description of equipment needed to perform the 1-mm skin biopsy, as well as cell culture and molecular biology techniques, can be found in the “Reagents and Materials” section.

This protocol was derived from a study reviewed and approved by the Comité de Ética de la Investigación de Cádiz (CEI Cádiz) (Protocol Code: FPS-CMER-2022; Registration No. 110.22) in accordance with the provisions of Spanish Law 14/2007, of July 3, on Biomedical Research and adheres to the General Data Protection Regulation (EU Regulation 2016/679, GDPR). Once the ethics committee approved the significant protocol modification on September 28, 2023, we took the first skin biopsy from our first control volunteer on October 25, 2023. The last sample included in the study was taken on August 1, 2025. Written informed consent was obtained from all participants prior to their inclusion in the study. By adhering to these internationally recognized guidelines, this protocol seeks to uphold the highest ethical standards in biomedical research while safeguarding the rights, dignity, and well-being of all participants.

## 3. Expected results

**Fig 1:**
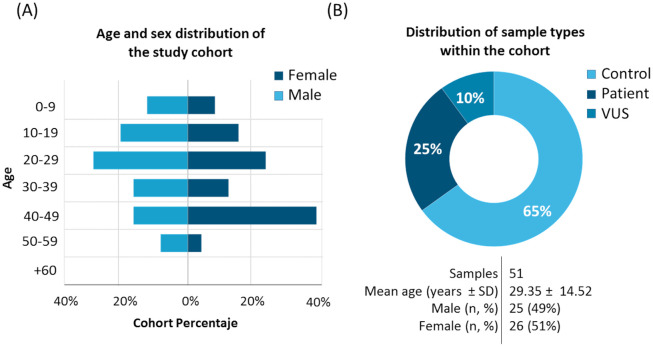
Demographic characteristics of the study cohort. The left graph represents the age distribution of participants by sex. The accompanying table summarizes the number of samples, sex distribution (% male and female), and mean ± SD of age. The right graph represents the health status distribution of participants.

The analyzed cohort is part of a study focused on the functional evaluation of genetic variants of uncertain significance (VUS) in pediatric patients harboring clinical phenotype of RASopathy and their family members. Accordingly, the current reduced-biopsy protocol was specifically designed to be minimally invasive and robustly suitable for this sensitive population. The study included 51 participants of both sexes and across a wide age range, comprising 65% healthy controls, 25% affected patients and 10% of VUS carriers (Fig 1). Despite the inherent clinical and genetic heterogeneity of this cohort, all HDFs cultures were successfully established and demonstrated high viability, validating the robustness and broad applicability of the developed protocol.

Skin biopsies were obtained from the upper right region of the back at subscapular level. This site was selected as it contains the thickest dermis [24] and is subjected to minimal mechanical stress, thereby minimizing post-procedural discomfort. Maintaining a consistent sampling site across individuals is essential to ensure reproducibility, given the presence of distinct fibroblast subtypes and transcriptional heterogeneity in different skin regions.

Across all applications of this method, no instances of infection, suture requirement, excessive bleeding, or specific wound care were observed or reported. Only one intervention required repetition due to sample contamination after overnight storage at 4ºC. Collectively, these findings demonstrate a 98% success rate and underscore the reliability of the protocol.

Two methodological changes have enhanced the risk-to-benefit ratio, thereby increasing its potential for routine clinical and research implementation. The first is the replacement of intradermal for topic anesthesia (e.g., EMLA). This modification is highly pertinent for pediatric and teenage donors, for whom needle phobia associated with routine medical procedures, such as vaccination or blood collection, is a frequent concern. Eliminating painful intradermal injections minimizes procedural distress, enhancing patient compliance and facilitating recruitment.

**Fig 2:**
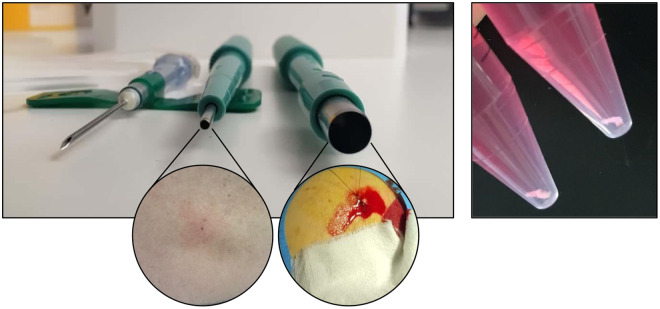
Comparison of biopsy punch size and handling of tissue explants. Left: Illustrative image showing the relative diameters of a standard blood collection needle, a 1-mm punch and a 4-mm punch. Corresponding skin wounds are visible at the bottom. Right: 15-ml conical Falcon tubes containing 1-mm TE prepared for culture.

The second refinement is the reduction of the biopsy punch size to 1 mm. This effectively addresses a major concern in conventional methods. The smaller punch, with a diameter comparable to a standard blood collection needle, reduces invasiveness and patient discomfort, eliminating the post-procedural sutures and wound management. The resulting reduced wound typically closes spontaneously within hours and is often imperceptible the following day. In contrast, biopsies obtained with 3–4 mm punches require stitches, more extensive wound care, are susceptible to infections, and frequently result in noticeable scarring (Fig 2, left).

Due to its minimal invasiveness, this procedure could be performed during routine follow-up visits by trained physicians at the point of care, reducing patient burden. Moreover, the associated protocol is straightforward, cost-effective, and feasible in any facility with basic cell culture equipment, promoting wide accessibility and scalability for biomedical studies.

Beyond improving the clinical feasibility of skin sampling, the introduction of the 1-mm punch also streamlined the subsequent cell culture process. In our opinion, the clean and uniform excision produced by the punch facilitates efficient adhesion of the TE to the culture surface, as straight and regular edges adhere more efficiently than irregular ones. Moreover, the soft and pliable nature of the dermal tissue, combined with the high surface area-to-volume ratio characteristic of the 1-mm TE, facilitates localized dehydration. This process generates strong capillary and surface tension forces that actively promote the stable adherence and slight deformation (flattening) of the cylindrical explant (Fig 2, right) against the bottom of the culture well, thereby maximizing the total tissue-to-surface contact area and promoting efficient cellular migration. Therefore, it is unnecessary to determine tissue orientation under a microscope during plating. This simplification further reduces handling time and plausibly, risk of contamination, making the protocol feasible in laboratories equipped with only basic cell culture facilities.

The 1-mm TE provided sufficient material for establishing HDFs cultures that reached confluence in a six-well plate within approximately one month, yielding sufficient cell mass for experimental applications or cryogenic preservation. Consistent with previous reports, the yield and proliferative capacity of the isolated HDFs in our cohort were influenced by inherent donor characteristics such as age and health status and individual genetic background [25]. While the expected variability was observed, such as a higher proliferative capacity in younger donors compared to older ones, these fluctuations remained within known biological ranges and did not induce notable deviations in the isolation and culture protocol execution. The robustness of the method, considering the clinical and genetic heterogeneity of the 51 donors, lends further credence to its validity.

**Fig 3:**
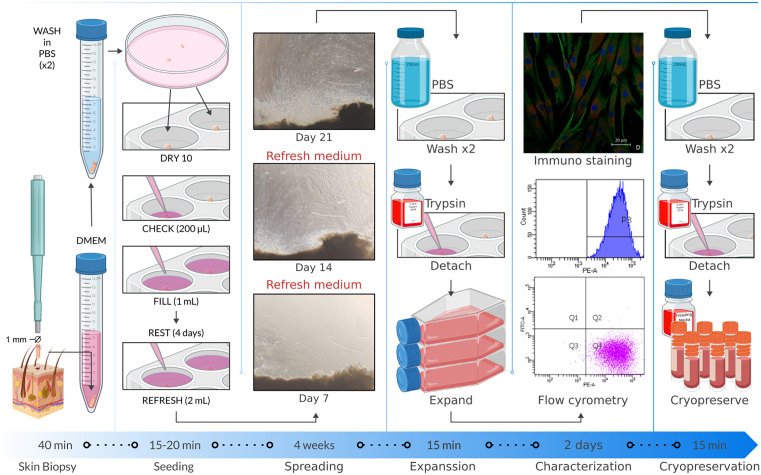
Summary of the protocol, including the four main steps: 1) biopsy extraction, 2) seeding and spreading, 3) expansion, and 4) cryopreservation; followed by the characterization of primary fibroblast.

To further validate the functionality and purity of the derived HDFs cultures, phenotypic characterization was performed through the analysis of growth kinetics, expression of fibroblast-specific markers by immunofluorescence, and profiling of cell populations across passages by flow-cytometry (Fig 3). These results are further detailed in the following sections.

### 3.1 Growth curves

Given the reduction in TE size to 1 mm, it was essential to verify whether the resulting HDFs cultures provided sufficient cells for downstream molecular biology applications and to assess whether they retained robust proliferative capacity and viability. To address these questions, we evaluated the cumulative population doubling level (PDL) and the growth kinetics of the derived HDFs cultures.

**Fig 4:**
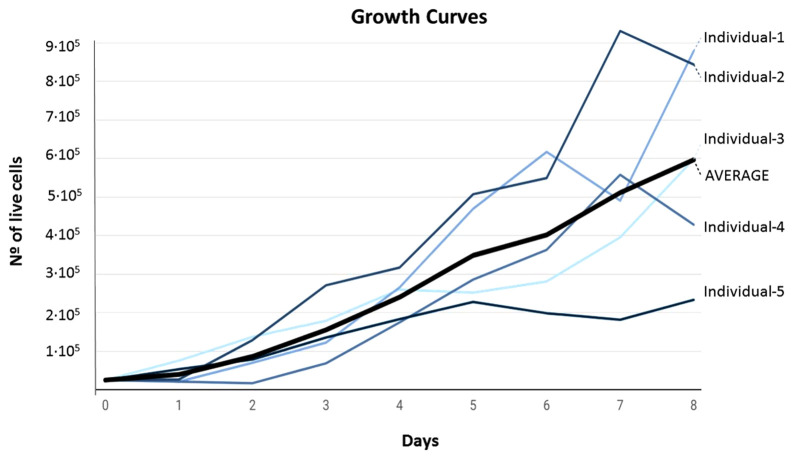
Growth curve of human fibroblasts obtained from five different individuals. Third-passage human fibroblast cultures were seeded in 24-well plates and maintained in complete DMEM. Two wells per individual were counted daily. The mean cell growth is shown as a black thicker line. During the first day after seeding, cell numbers tended to remain constant, indicating a short lag phase. From the second day, cells entered an exponential growth phase lasting until day 6, reaching a PDL of 3.2, corresponding to an average doubling time of approximately 1.5 days.

We estimated the cumulative PDL based on standard flask capacities and culture progression. Each HDFs line typically underwent four expansion cycles following the sequence [TE → 1xT25 → 1xT75 → 3xT75 → cryopreservation]. Considering an estimated confluence density of 2.5·10^5^ cells per explant and approximately 5·10^6^ cells per T75 flask, the theoretical cumulative yield across these passages would be at least 6·10^7^ cells. Using the standard formula for population doublings (N_H_/N_I_ = 2^X^, where N_I_ is the inoculum number, N_H_ the harvested cell number, and x the population doubling level), this corresponds to a minimum cumulative PDL of approximately eight. These estimates indicate that even from a 1-mm skin biopsy, sufficient fibroblasts may be expanded for multiple experimental assays and/or cryogenic storage. Cultured fibroblasts experience progressive gene expression changes with increasing passage number. Therefore, some considerations should be taken into account before adopting a protocol requiring 4 passages, depending on the intended application. Specifically, studies aimed at characterizing cellular identity and population heterogeneity by scRNA-seq, or at epigenomic profiling of the original tissue, should be performed on ex vivo tissue or at passage 1 at the most, as culture-induced transcriptional convergence and epigenetic drift have been shown to occur even at early passages [26,27]. However, for the majority of molecular biology applications, passage 4 remains within an acceptable range, as the main documented confounding factors — widespread replicative senescence and cumulative epigenetic drift — have not yet manifested to a substantial degree at this stage, according to the available evidence [26,28].To experimentally confirm fibroblast growth and viability, third-passage fibroblasts were seeded at 2,5·10^4^ cells per cm^2^ in 24-well culture plates and maintained in DMEM supplemented with 20% FBS. Two wells per individual were harvested and counted daily over 8 days using Trypan Blue staining and a Bio-Rad TC20™ Automated Cell Counter. The cultures exhibited a lag phase followed by robust proliferation, with an average doubling time of 1.5 days during the exponential growth phase (days 2–6), consistent with the natural growth observed in HDFs cultures [29] (Fig 4). Cell viability remained high throughout the experiment (average of viable cells = 92.1% ± 2.5), indicating that HDFs maintained functionality without evidence of early senescence, supporting their applicability for downstream molecular analyses within the standard passage range.

### 3.2 Immunofluorescence

**Fig 5:**
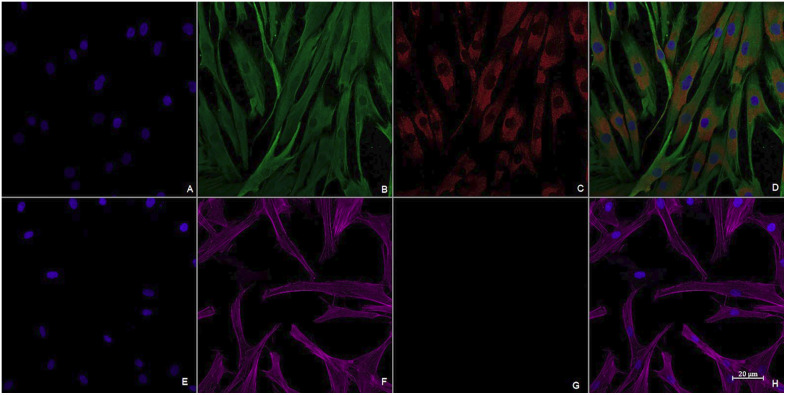
Characterization of HDFs derived from skin biopsy by immunofluorescence. Nuclei were counterstained by DAPI **(A, E)**. Fibroblast-specific markers: anti-vimentin (green) **(B)** and anti-human fibroblast surface protein (red) **(C)**. Merged images A-C **(D)**. F-actin detected with Phalloidin-iFluorTM 647 conjugate (purple) **(F)**. Fibroblast negative marker EpCAM/TROP. **(G)** Merged images E-G **(H)**. Images were acquired with 20x magnification.

The identity of HDFs was assessed by immunofluorescence, using vimentin and human fibroblast surface protein as positive markers and EpCAM/TROP as a negative marker (Fig 5). HDFs were seeded in 12-mm poly-D-lysine coated coverslips, fixed with paraformaldehyde 4% for 15 minutes and permeabilized with 0.1% Triton X-100 for 10 minutes. Primary antibodies were incubated overnight at 4ºC. After thoroughly washing, secondary antibodies were applied for 2 hours and nuclei were counterstained with DAPI. Coverslips were mounted using Vectashield, and images were acquired using a ZEISS LSM 900 confocal microscope. Phalloidin staining revealed the characteristic spindle-shaped morphology of HDFs. Images confirmed that cells were positive for fibroblast-specific markers, with a high signal from vimentin and human fibroblast surface protein. Moreover, cells were negative for EpCAM/TROP, confirming the absence of epithelial contamination and validating the fibroblast identity and purity of the culture.

### 3.3 Flow cytometry

**Fig 6:**
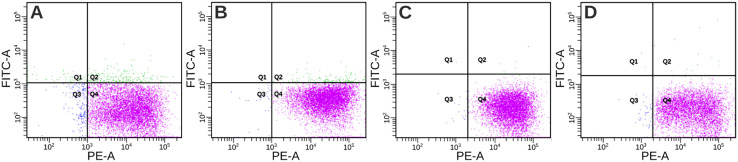
Phenotypic characterization of cell populations derived from skin biopsies across passages 0-3. Experiments were performed on five different donors. **(A-D)** Representative flow cytometry dot plots showing cell populations positive for CD49f (FITC-A) and S100A4 (PE-A). The mean percentages (± SD) of S100A4 + cells were 87.76 ± 14.07% in the cell mass derived from the biopsy **(A)**, 91.52 ± 4.96% in passage 1 **(B)**, 99.5 ± 0.23% in passage 2 and 97.84 ± 1.25% **(C)** in passage 3 **(D)**. Each dot plot corresponds to the most representative sample for the respective passage; therefore, fluorescence thresholds may differ among panels, as they were independently determined for each donor based on its corresponding negative control.

To ensure the purity of the HDFs primary culture, we conducted flow cytometry analysis, acknowledging that the initial skin biopsy may contain other cell types, including keratinocytes, melanocytes, and immune cells such as lymphocytes or macrophages. We tracked the evolution of cellular subpopulations from the tissue explant stage through successive passages, achieving >99% fibroblast purity by passage 3. At the biopsy stage, cells were passaged upon reaching 100% confluence as described in the online protocol [23], with 1 × 10⁶ cells reserved for flow cytometry analysis using a BD FACSCelesta™ SORP cytometer. Monoclonal antibodies S100A4-PE and integrin α6-FITC were employed as positive and negative fibroblast markers, respectively, following 15 minutes fixation with PFA 4% and 30 minutes permeabilization with 0,2% Triton X-100. After washing, the cellular mass was predominantly made up of fibroblasts, with ~88% of cells positive for S100A4. Over successive passages, HDFs purity progressively increased, reaching >99% by passage 3 (Fig 6). These findings confirmed that the culture conditions effectively favored HDFs enrichment while minimizing contamination from other cell types.

## Supporting information

S1 File A detailed step-by-step protocol. (PDF)

S2 File A detailed list of reagents and materials. (ODT)

S3 File (PDF)

## References

[1] DeRoin L, Cavalcante De Andrade Silva M, Petras K, Arndt K, Phillips N, Wanjari P, et al. Assessing the feasibility and limitations of cultured skin fibroblasts for germline genetic testing in hematologic disorders. Blood. 2020;136(Supplement 1):35–6. doi: 10.1182/blood-2020-138431 PMC9177640 35419889

[2] Guo D, Zhang L, Wang X, Zheng J, Lin S. Establishment methods and research progress of livestock and poultry immortalized cell lines: a review. Front Vet Sci. 2022;9:956357. doi: 10.3389/fvets.2022.956357 36118350 PMC9478797

[3] Cerneckis J, Cai H, Shi Y. Induced pluripotent stem cells (iPSCs): molecular mechanisms of induction and applications. Signal Transduct Target Ther. 2024;9(1):112. doi: 10.1038/s41392-024-01809-0 38670977 PMC11053163

[4] Khoo TS, Jamal R, Abdul Ghani NA, Alauddin H, Hussin NH, Abdul Murad NA. Retention of somatic memory associated with cell identity, age and metabolism in induced pluripotent stem (iPS) cells reprogramming. Stem Cell Rev Rep. 2020;16:251–61. 32016780 10.1007/s12015-020-09956-x

[5] Poetsch MS, Strano A, Guan K. Human induced pluripotent stem cells: from cell origin, genomic stability, and epigenetic memory to translational medicine. Stem Cells. 2022;40(6):546–55. doi: 10.1093/stmcls/sxac020 35291013 PMC9216482

[6] Hayflick L, Moorhead PS. The serial cultivation of human diploid cell strains. Exp Cell Res. 1961;25:585–621. doi: 10.1016/0014-4827(61)90192-6 13905658

[7] Alberts B, Heald R, Johnson A, Morgan D, Raff MC, Roberts K, et al. Molecular biology of the cell. W. W. Norton; 2022.

[8] Deng C-C, Hu Y-F, Zhu D-H, Cheng Q, Gu J-J, Feng Q-L, et al. Single-cell RNA-seq reveals fibroblast heterogeneity and increased mesenchymal fibroblasts in human fibrotic skin diseases. Nat Commun. 2021;12(1):3709. doi: 10.1038/s41467-021-24110-y 34140509 PMC8211847

[9] Driskell RR, Watt FM. Understanding fibroblast heterogeneity in the skin. Trends Cell Biol. 2015;25(2):92–9. doi: 10.1016/j.tcb.2014.10.001 25455110

[10] Lynch MD, Watt FM. Fibroblast heterogeneity: implications for human disease. J Clin Invest. 2018;128(1):26–35. doi: 10.1172/JCI93555 29293096 PMC5749540

[11] Bensa T, Tekkela S, Rognoni E. Skin fibroblast functional heterogeneity in health and disease. J Pathol. 2023;260(5):609–20. doi: 10.1002/path.6159 37553730

[12] Liu K, Cui Y, Han H, Guo E, Shi X, Xiong K, et al. Fibroblast atlas: shared and specific cell types across tissues. Sci Adv. 2025;11(14):eado0173. doi: 10.1126/sciadv.ado0173 40173240 PMC11963979

[13] Philippeos C, Telerman SB, Oulès B, Pisco AO, Shaw TJ, Elgueta R. Spatial and single-cell transcriptional profiling identifies functionally distinct human dermal fibroblast subpopulations. J Invest Dermatol. 2018;138:811–25. 29391249 10.1016/j.jid.2018.01.016 PMC5869055

[14] Zhu X, Kang H, Zhao Z, Jia P. A systematic characterization of fibroblast subtypes and heterogeneity. iScience. 2025;28(12):113915. doi: 10.1016/j.isci.2025.113915 41377662 PMC12686731

[15] GTEx Consortium. The GTEx Consortium atlas of genetic regulatory effects across human tissues. Science. 2020;369(6509):1318–30. doi: 10.1126/science.aaz1776 32913098 PMC7737656

[16] UniProt Consortium. UniProt: the Universal Protein Knowledgebase in 2025. Nucleic Acids Res. 2025;53:D609–17. 10.1093/nar/gkae1010 PMC11701636 39552041

[17] Villegas J, McPhaul M. Establishment and culture of human skin fibroblasts. Curr Protoc Mol Biol. 2005;Chapter 28:Unit 28.3. 10.1002/0471142727.mb2803s71 18265368

[18] Kim SH, Kim JH, Lee SJ, Jung MS, Jeong DH, Lee KH. Minimally invasive skin sampling and transcriptome analysis using microneedles for skin type biomarker research. Skin Res Technol. 2022;28(2):322–35. doi: 10.1111/srt.13135 35007372 PMC9907599

[19] Wang CY, Maibach HI. Why minimally invasive skin sampling techniques? A bright scientific future. Cutan Ocul Toxicol. 2011;30(1):1–6. doi: 10.3109/15569527.2010.517230 20883150

[20] Ziliani F, Michalak-Micka K, Klar AS. Isolation and culture of human dermal fibroblasts. Methods Mol Biol. 2025;2922:75–83. doi: 10.1007/978-1-0716-4510-9_6 40208528

[21] Vangipuram M, Ting D, Kim S, Diaz R, Schüle B. Skin punch biopsy explant culture for derivation of primary human fibroblasts. J Vis Exp. 2013;2013:e3779. doi: 10.3791/3779 PMC3731437 23852182

[22] Murillo-Pineda M, Martínez-Miralles J, Medina-Calzada Z, Varela RM, Macías FA, Chinchilla N, et al. New insights into the molecular actions of grosheimin, costunolide, and α- and β-cyclocostunolide on primary cilia structure and hedgehog signaling. Int J Mol Sci. 2025;26(23):11754. doi: 10.3390/ijms262311754 41373900 PMC12692604

[23] Heredia-Torrejón M, Puga-López B, Guerrero-López M, Lechuga-Sancho M, Montañez R. A minimally invasive lab protocol for fibroblasts isolation from 1-mm skin punch biopsies in pediatric patients v2. 2024. 10.17504/protocols.io.81wgbrrjylpk/v2 PMC13286191 42329873

[24] Olsen LO, Takiwaki H, Serup J. High-frequency ultrasound characterization of normal skin. Skin thickness and echographic density of 22 anatomical sites. Skin Res Technol. 1995;1(2):74–80. doi: 10.1111/j.1600-0846.1995.tb00021.x 27328386

[25] Kisiel MA, Klar AS. Isolation and culture of human dermal fibroblasts. Methods Mol Biol. 2019;1993:71–8. doi: 10.1007/978-1-4939-9473-1_6 31148079

[26] Matsuyama M, WuWong DJ, Horvath S, Matsuyama S. Epigenetic clock analysis of human fibroblasts in vitro: effects of hypoxia, donor age, and expression of hTERT and SV40 largeT. Aging (Albany NY). 2019;11(10):3012–22. doi: 10.18632/aging.101955 31113906 PMC6555444

[27] Lock A, Drudi EM, Freydina D, Stramer BM, Denk F, Shaw TJ. Single-cell RNA sequencing clarifies dermal fibroblast subset representation in vitro and reveals variable persistence of keloid disease-associated features. BioRxiv. 2025. doi: 10.1101/2025.08.11.668109

[28] Schäuble S, Klement K, Marthandan S, Münch S, Heiland I, Schuster S, et al. Quantitative model of cell cycle arrest and cellular senescence in primary human fibroblasts. PLoS One. 2012;7(8):e42150. doi: 10.1371/journal.pone.0042150 22879912 PMC3413708

[29] Hadjipanayi E, Mudera V, Brown RA. Close dependence of fibroblast proliferation on collagen scaffold matrix stiffness. J Tissue Eng Regen Med. 2009;3(2):77–84. doi: 10.1002/term.136 19051218

