# Supplementary material for: A minimally invasive lab protocol for fibroblasts isolation from 1-mm skin punch biopsies in pediatric patients
Source: PLoS One. 2026 Jun 22;21(6):e0350828. doi: 10.1371/journal.pone.0350828 (PMC13286191; doi:10.1371/journal.pone.0350828)
Supplement: S3 File — (PDF) [file pone.0350828.s003.pdf]

Mar 03, 2025 Version 2

A Minimally Invasive Lab Protocol for Fibroblasts Isolation from 1-mm Skin Punch Biopsies in Pediatric Patients V.2

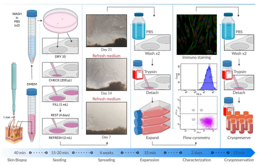

DOI

https://dx.doi.org/10.17504/protocols.io.81wgbrrjylpk/v2

María eredia-Torrejón[1], Begoña uga-López[2], Dolores M. Guerrero-López[2], Alfonso M. Lechuga-Sancho[1,3], Raúl Montañez[4]

[1]Department of Child and Mother Health and Radiology, Instituto de Investigación e Innovación Biomédica de Cádiz (INiBICA), Medical School, Universidad de Cádiz, Cádiz, Spain.;
[2]Research Unit, Instituto de Investigación e Innovación Biomédica de Cádiz (INiBICA), Hospital Universitario Puerta del Mar, Universidad de Cádiz, Cádiz, Spain.;
[3]Division of Endocrinology, Department of Pediatrics, Hospital Universitario Puerta del Mar, Universidad de Cádiz, Instituto de Investigación e Innovación Biomédica de Cádiz (INiBICA), Cádiz, Spain.;
[4]Department of Molecular Biology and Biochemistry, University of Málaga, Andalucía Tech, E-29071 Málaga, Spain.

María eredia-Torrejón: These authors contributed equally;
Begoña uga-López: These authors contributed equally;
Raúl Montañez: Correspondence: raulemm@uma.es;

Raúl RMM Montañez
Universidad de Málaga

Create & collaborate more with a free account

Edit and publish protocols, collaborate in communities, share insights through comments, and track progress with run records.

Create free account

OPEN ACCESS

DOI: https://dx.doi.org/10.17504/protocols.io.81wgbrrjylpk/v2

Protocol Citation: María eredia-Torrejón, Begoña uga-López, Dolores M. Guerrero-López, Alfonso M. Lechuga-Sancho, Raúl Montañez 2025. A Minimally Invasive Lab Protocol for Fibroblasts Isolation from 1-mm Skin Punch Biopsies in Pediatric Patients. **protocols.io https://dx.doi.org/10.17504/protocols.io.81wgbrrjylpk/v2**Version created by **Raúl RMM Montañez**

License: This is an open access protocol distributed under the terms of the **Creative Commons Attribution License**, which permits unrestricted use, distribution, and reproduction in any medium, provided the original author and source are credited

Protocol status: Working
We use this protocol and it's working

Created: March 03, 2025

Last Modified: March 03, 2025

Protocol Integer ID: 123676

Keywords: Cell culture, Genetic diseases, VUS, Primary Fibroblasts, Pediatric patients, Skin biopsy, Fibroblasts isolation, minimally invasive lab protocol for fibroblasts isolation, mm skin punch biopsies in pediatric patient, fibroblasts from skin biopsy, mm skin punch biopsy, fibroblasts isolation, isolating fibroblast, skin biopsy, pediatric variants of uncertain significance, punch biopsy diameter, purity of isolated fibroblast, isolated fibroblast, interpreting pediatric variant, diagnosis of rare genetic disease, meaningful advancement in pediatric research, molecular diagnostics laboratory, pediatric research, patients with rare genetic disorder, study of rare genetic disease, minimally invasive lab protocol, analyzing molecular phenotype, large tissue sample, rare genetic disease, rare genetic disorder, suited for molecular diagnostics laboratory, pediatric patient, molecular phenotype, therapeutic study, invasive procedure, advanced diagnostic, enabling advanced diagnostic, diagnosis

Funders Acknowledgements:
Andalusian Ministry of Health and Families through the initiative (RPS 24664)
Grant ID: PI-0069-2022

Abstract

The integration of multi-omics techniques has revolutionized the diagnosis of rare genetic diseases. However, interpreting pediatric variants of uncertain significance (VUS) remains a significant challenge. Skin biopsies offer a valuable source of culturable cells for analyzing molecular phenotypes by functional and omic studies, especially in patients with rare genetic disorders. Traditional methods for isolating fibroblasts from skin biopsies often require large tissue samples, invasive procedures, and subsequent wound care, which can be particularly distressing for pediatric patients and deviates from ethical standards. To overcome this limitation, we developed a simplified protocol that minimizes patient discomfort. By reducing the punch biopsy diameter to as little as 1 mm, we significantly reduce invasiveness while achieving high yield and purity of isolated fibroblasts. This streamlined, minimally invasive approach is well-suited for molecular diagnostics laboratories and facilitates the study of rare genetic diseases in children. By enabling advanced diagnostics, therapeutic studies, and personalized medicine, this protocol represents a meaningful advancement in pediatric research and clinical care.

Image Attribution

Figure 1: Raúl Montañez; Figure 2: María Heredia-TOrrejón and Begoña Puga-López; Figure 3 and 4 Mª Dolores Guerrero-López.

Guidelines

Biological samples collected as part of this protocol will be used exclusively for the purposes defined in the protocol. In accordance with international ethical guidelines, samples will only be retained as long as necessary for the research and will be destroyed upon the protocol's conclusion unless additional consent is obtained for their further use.
Patients will be assigned anonymized identification codes for all records to ensure that their clinical data cannot be linked to any identified or identifiable individual. Re-identification of participants will only be possible under exceptional circumstances and for strictly clinical reasons, as authorized by the designated principal investigator.

Materials

	A	B	C
	**Reagents and Materials**	**Source**	**Reference**
	Cell culture		
	DMEM low glucose	PanBiotech	Cat. No.: P04-01550
	Fetal Bovine Serum (FBS)	Corning	Ref: 35-079-CV
	Penicillin-Streptomycin	PanBiotech	Cat. No.: P06-07100
	0.25% Trypsin/EDTA	PanBiotech	Cat. No.: P10-019500
	Dimethylsulfoxide (DMSO) for cell culture	PanReac AppliChem	A3672
	15 mL conical falcon	Deltalab	429942
	22-cm2 Petri dish	Soria Genlab	P101
	6-well culture plate	Sarstedt	83.3920
	24-well culture plate	Sarstedt	83.3922
	Sterile 12 mm glass coverslip	Labolan	20012
	5 mL Polystyrene Round-Bottom Tube	Corning	352052
	Poly-D-lysine	Sigma-Aldrich	P1024
	Immunofluorescence		

	A	B	C
	Monoclonal Anti-Human Fibroblast Surface Protein (1:400)	Sigma	F4771
	Vimentin (1:500)	NovusBio	NB300-223
	EpCAM/TROP-16	Biotechne (R&D System)	AF960
	Goat anti-Mouse IgG (H+L) Cross Adsorbed Secondary Antibody, Alexa Fluor TM 568 (1:1000)	Invitrogen	#A-11004
	Goat anti-Chicken IgY (H+L) Cross Adsorbed Secondary Antibody, Alexa Fluor TM 488 (1:1000)	Invitrogen	#A32931
	Donkey Anti-Goat IgG H&L (Alexa Fluor 750) (1:1000)	abcam	ab175744
	Phalloidin-iFluorTM 647 conjugate	Cayman Chemical	20555
	DAPI	Santa Cruz	sc-3598
	Flow cytometry		
	CD49f Antibody, Anti-human/mouse, FITC	Miltenyi Biotec	130-097-245
	PE anti-human,	BioLegend	NJ-4F3-D1

	A	B	C
	S100A4 Antibody		
	Skin Biopsy		
	Biopsy Punch 1 mm	Kai Medical	BP-10F
	EMLA (Anesthetic cream)	Aspen	#679290
	Prontosan (Antiseptic)	Braun	#400403
	Adhesive bandage	IHT	0037107
	Sterile gauze	Texpol	1186.01
	Gloves	Biomedica	140158
	Sterile tweezers	Medical Waitch	Ref.166
	Sterile scalpel	Medical Waitch	Ref.24
	Equipment		
	Laminar Flow Hood	Holten LaminAir	Model 1.2
	Water bath, 37ºC	Trade Raypa	
	Incubator 37ºC, 5% CO2	Thermo Scientific	HeraCell 150
	Centrifuge	Eppendorf	5810R
	Confocal Microscope	Zeiss	Axio Observer
	Inverted Phase-Contrast Microscope	Olympus	CKX41
	Cytometer, FACS CELESTA SORP	Becton Dickinson	

Protocol materials

1X PBS (Phosphate-buffered saline)

Safety warnings

The application of this protocol to pediatric patients requires specific ethical considerations to ensure their rights, dignity, and well-being are protected. The following measures must be strictly adhered to:

1. Informed Consent and AssentInformation for Legal Guardians: Comprehensive and clear information about the purpose, procedures, potential risks, and benefits of the protocol must be provided to the child's parents or legal guardians. This information should include how data and biological samples will be used, stored, and disposed of.
Adapted Information for the Child: The child must also receive information about the procedure in a manner appropriate to their age, maturity, and level of understanding. This should include explanations of the steps involved, reassurance about their safety, and the opportunity to ask questions or express concerns.
Consent and Assent: While the formal consent will be obtained from the legal guardians, the child's assent (agreement) must also be sought, respecting their capacity to understand and their willingness to participate.

2. Minimizing Psychological Stress

- **Preparation for the Procedure**: Physicians or nurses performing the biopsy must follow an adapted protocol aimed at reducing the child's anxiety and discomfort. This includes:

1. Explaining the procedure in a calm and friendly manner.
2. Using child-friendly language and visuals if necessary.
3. Allowing the child to familiarize themselves with the medical environment and tools beforehand.

- **Use of Distraction Techniques**: During the procedure, distraction strategies such as toys, videos, or music may be used to divert the child's attention from the biopsy and alleviate fear or distress.
- **Presence of a Comforting Figure**: A parent, guardian, or a familiar caregiver should be present to provide emotional support to the child throughout the procedure.

3. Pain Management

- **Local Anesthesia**: Adequate pain relief measures must be applied, such as local anesthesia, to minimize physical discomfort.
- **Observation of Stress Responses**: The attending healthcare professional must monitor the child for signs of distress and take appropriate steps to address them promptly.

4. Post-Procedure Care

- **Emotional Support**: After the biopsy, children should be provided with reassurance and positive reinforcement to reduce lingering anxiety or fear of future medical procedures.
- **Follow-Up Communication**: Both the child and their guardians should be informed about the outcome of the procedure in an age-appropriate and clear manner.

5. Ethical Responsibility

The healthcare team must prioritize the child's psychological and physical comfort at all times. The implementation of this protocol must adhere to the principles of **non-maleficence** (avoiding harm),

beneficence (acting in the best interest of the patient), and **respect for autonomy** (acknowledging the child's evolving capacity for decision-making).

By implementing these measures, the protocol aims to ensure that children participating in the research are treated with the utmost care and respect, minimizing potential distress and safeguarding their well-being.

Ethics statement

This protocol will adhere to the ethical principles set forth in the **Declaration of Helsinki** (2013, Fortaleza revision) by the World Medical Association, which provides guidelines for medical research involving human subjects, including respect for individuals, informed consent, and the safeguarding of participants' rights and welfare.
Additionally, it will align with the **International Ethical Guidelines for Biomedical Research Involving Human Subjects** issued by the Council for International Organizations of Medical Sciences (CIOMS) in collaboration with the World Health Organization (WHO). These guidelines address ethical considerations such as confidentiality, informed consent, and the ethical review of research protocols.
The protocol will undergo review and approval by a recognized **Research Ethics Committee** to ensure compliance with these international ethical standards.
The collection, processing, and storage of personal data, as well as biological samples, have to comply with the principles of data protection and privacy outlined in the **General Data Protection Regulation (EU Regulation 2016/679, GDPR)** and relevant international standards. Reasonable and appropriate measures will be implemented to safeguard patient data and maintain confidentiality. Personal information will be securely encoded to prevent identification, and only authorized personnel performing professional duties will have access to identifiable data.
Participants will have the right to exercise their rights to access, rectify, or request the deletion of their data, as outlined in international data protection standards. Procedures will be in place to address these requests promptly and in accordance with the conditions of the protocol and applicable ethical and legal requirements.
By adhering to these internationally recognized guidelines, this protocol seeks to uphold the highest ethical standards in biomedical research while safeguarding the rights, dignity, and well-being of all participants.

Before start

Informed consent will be obtained from all participants or, in the case of minors, from their parents or legal guardians. This consent process will ensure participants and their representatives receive comprehensive and understandable information about the objectives, procedures, and potential risks and benefits of the protocol. They will also be informed about their rights to withdraw from the protocol at any time without any adverse consequences.

Introduction

1 The field of biomedical research has entered the “omics” era, marked by a surge in our capacity to mine molecular information. The integration of multi-omics techniques offers unprecedented power to explore genomic anomalies and regulatory alterations [1], far beyond the limitation of the restricted set of targets we had a decade ago. These technological improvements have enhanced our diagnostic yield in patients with rare genetic diseases, where clinical diagnosis alone does not suffice due to phenotypic overlap among different conditions, often resulting in misdiagnoses [2]. A correct molecular diagnosis is critical for establishing the natural course of a disease, therefore tailoring an optimal management plan. It empowers clinicians to implement preventive measures, monitor disease progression and manage symptoms effectively, ultimately leading to personalized therapies and better patient outcomes. Additionally, a confirmed diagnosis alleviates anxiety for patients and their families, facilitating informed decision-making through genetic counseling [3].

However, a significant challenge remains in this diagnostic process: data interpretation. Advances in omics techniques enable exploration of organizational and regulatory complexity across multiple levels. For instance, next-generation sequencing (NGS) techniques give us the means to explore entire genome sequences, but they also involve the interpretation of an average of 5·106 variants [4]. This limitation in data integration and interpretation, often left genetic testing to identify candidate variants classified as variants of uncertain significance (VUS). These variants hinder the establishment of a definitive diagnosis, becoming a serious problem that worsens as more genomes are sequenced. For instance, in the case of Noonan Syndrome, up to 65% of variants in PTPN11 and up to 87,6% in SOS1 are classified as VUS [5]. These VUS require further evidence to be reclassified as “benign/likely benign” or “pathogenic/likely pathogenic” [6]. However, every VUS reclassified will benefit other patients harboring this variant. To overcome this limitation and achieve a definitive diagnosis, cells derived from skin biopsy emerge as an invaluable tool to analyze cellular and molecular phenotypes and gather experimental evidence to carry out this reclassification.

The limited availability of statistical evidence and specialized expertise, inherently present within the context of RDs, highlights the critical need for reclassifying VUS to improve patient care [7]. Patient-derived cell cultures offer a powerful tool for achieving these functional validations. However, traditional skin biopsy protocols (requiring 3–4 mm biopsy punches) are invasive, painful, and associated with significant risks such as bleeding, infection, and the need for sutures and wound care [8]. Recent advancements in biopsy techniques have introduced smaller 1 mm punches, which, being only marginally thicker than standard blood collection needles,

present a less painful and non-invasive alternative [9,10]. While these smaller samples are not suitable for anatomopathological studies, they suffice for establishing primary cultures critical for molecular biology research. This transition to minimally invasive methods greatly enhances patient comfort and reduces psychological distress, particularly in pediatric populations. Our optimized protocol successfully isolates fibroblasts from 1 mm skin tissue explants (TE), ensuring ethical and patient-friendly research practices. To facilitate widespread adoption in clinical laboratories, we simplified the protocol, reducing procedural complexity and contamination risks, thereby promoting its utility for Bed-to-Bench studies and translational research.

Over 32 applications of this method, we have observed no infections, need for sutures, or specific wound care requirements, with only one instance requiring a procedural repeat due to initial cell growth failure. These data underscore the efficiency, reliability, and potential for routine clinical integration of the proposed protocol.

Procedure

2h 15m

2 This protocol builds upon well-established practices for fibroblast isolation from skin biopsies [8,11], refining them to incorporate the minimally invasive 1 mm punch biopsy technique. While the suggested method offers a significant improvement over traditional approaches, it remains invasive and unpleasant, particularly for young children. Therefore, the benefits must be carefully weighed against the risks for patients. The protocol was approved by the local institutional ethical committee (Code# FPS-CMER-2022), ensuring adherence to ethical guidelines. Additionally, informed consent was obtained from all participants, and/or their legal guardians, ensuring they fully understood the nature of the procedure and any potential risks involved. To ensure the safety and reliability of the procedure, strict sterile techniques must be adhered to at all times to minimize the risk of pathogen transmission.

3 **STEP 1: OBTAINING A TISSUE EXPLANT (TE)**

40m

Considerations

1) Consistent biopsy site selection across patients is crucial to ensure reliable and comparable results, as cellular composition and gene expression can vary significantly between skin regions. We chose the area between the lower scapula and the spine for its accessibility, minimal patient discomfort, and thicker dermis, which yields sufficient tissue for analysis. Additionally, this dorsal skin originates from the somitic mesoderm, aligning it developmentally with chondrocytes.
2) Handle the skin biopsy with care to avoid contact with materials that could cause fixation, such as ethanol on tweezers or Prontosan on the skin or gauze.
3) Utilizing disposable biopsy punches with a plunger (Kai Medical; Japan), minimizes tissue handling, reduces the risk of contamination, and prevents the loss of the tissue cylinder.

4) When immediate culture is not possible, the sample may be stored at room temperature for up to 3-4 hours. For extended storage of up to 24 h, or during transport to another laboratory, the sample should be kept at 4ºC in the same conical tube used for collection. [12].

3.1 **Procedure:**

1. Prepare a 15 mL conical tube with 10 mL of fibroblast medium (**FBM**) (DMEM Low Glucose **Pan-Biotech Catalog** #P04-01550 , 20% Fetal Bovine Serum **Corning Catalog** #35-079-CV , 100 U/mL Penicillin **Pan-Biotech Catalog** #P06-07100 , 100 ng/mL Streptomycin **Pan-Biotech Catalog** #P06-07100) tempered to 37 °C .
2. Thinly apply EMLA **Aspen Surgical Catalog** #679290 to the desired area and allow it to act for at least half an hour.
3. Using a sterile gauze, cleanse the skin with a commercially available solution of purified water, betaine surfactant and 0.1% Polyaminopropyl Biguanide, Prontosan **B.Braun Medical Inc Catalog** #400403 .
4. Pinch the skin surrounding the biopsy site using the thumb and index finger. Carefully, punch down the 1-mm trocar with a rotating movement through the epidermis and dermis.

Equipment

1-mm Biopsy Punch	NAME
Biopsy Punches Plunger Type	BRAND
BPP-10F	SKU
https://kai-europe.com/medical/biopsypunches_plungertype.php?lang=en	LINK

1. After the extraction, clean the area with a sterile gauze soaked in Prontosan **B.Braun Medical Inc Catalog** #400403 and apply an adhesive bandage.
2. Transfer the sample into the conical tube with 10 ml of **FBM**, ensuring it settles at the bottom.

4 **STEP 2: PLATING THE TISSUE EXPLANT** 1h

The purpose of this step is to plate the skin explant in a culture dish to establish a primary cell line (**Figure 1**). Ensuring the **TE** adheres properly to the plate surface is critical, as poor adhesion will prevent fibroblast proliferation.

Considerations:

1. **Re-adhesion of Detached Tissue:** If the **TE** detaches, re-adhesion can be facilitated using a sterile 12 mm glass coverslip coated with Poli-D-lisina hydrobromide **Merck MilliporeSigma (Sigma-Aldrich) Catalog #**P1024 . Gently press the coverslip against the well's surface to secure the tissue, taking care to prevent breakage. Alternatively, a needle may be used as described by [12]. However, both methods can interfere with subsequent fibroblast trypsinization and should only be employed when absolutely necessary.
2. **Medium Management for Adhesion:** Initially, add 1 mL of medium to facilitate **TE** adhesion, ensuring the tissue is fully submerged and does not float. Regularly inspect the well to confirm that the **TE** remains consistently covered with medium.
3. **Fibroblast Growth Observation:** The emergence of fibroblasts from the **TE** varies in timing, but growth is typically observed around one-week post-plating. After 5–7 days, keratinocytes will typically sprout from the epidermis and fibroblasts from the dermis. Use an inverted phase-contrast microscope to monitor the culture until the fibroblasts reach the desired confluence. Throughout this period, the fibroblast medium must be replaced with fresh medium every week.

4.1 **Preparation:**

- Sterilize the laminar flow hood with UV light for at least 30 minutes before use.
- Thoroughly clean the hood and set up materials using sterile techniques.
- Pre-warm 1X PBS (Phosphate-buffered saline) and **FBM** to 37ºC in a water bath.
- Place a 6-well plate in the incubator to temper.

4.2 **Preprocessing the TE:**

- Discard the medium from the **TE** tube by decantation, and gently wash the sample twice with warm 1X PBS (Phosphate-buffered saline) to eliminate fibers and contaminants.
- Add 1 mL of warm **FBM** to the lid of a 22-cm² Petri dish.
- Transfer the **TE** using sterile tweezers and place it into the medium. Split the **TE** horizontally into two halves with a sterile scalpel.

4.3 **Plating the TE Halves:**

- Place each half in a separate well of the tempered 6-well plate using tweezers. Drag the **TE** pieces over the well to improve adhesion by removing excess medium.
- Tilt the plate and wait 10–15 minutes for the residual medium to evaporate, ensuring proper adherence. The formation of a "halo" around the fragment indicates

successful adhesion.

- Carefully add 200 µL of **FBM** to each **TE**, ensuring the pieces remain submerged and adhered. Once adhesion is verified, gently add 1 mL of **FBM** to soak the entire surface.
- Place the plate in a 37ºC, 5% CO_2 incubator. Avoid disturbing the plate for 4 days to prevent detachment. Afterward, add up to 2 mL of **FBM** to each well.

5 **STEP 3: Establishment and maintenance of fibroblast primary cell line** 15m

Once fibroblasts cover approximately 30–40% of the well, the culture is ready for expansion, typically occurring 3–4 weeks after the tissue explant (TE) is plated. Each TE generally yields an average of four 25-cm² flasks. Passage 0 fibroblasts derived from these explants can be expanded through approximately 8–10 passages before reaching the Hayflick limit [13]. This expansion capacity ensures the production of sufficient cells for experimental needs while enabling the cryopreservation of an adequate number of vials for future validation. As the biopsy undergoes trypsinization and the extracellular matrix is digested, new passage 0 fibroblasts emerge, facilitating continued fibroblast isolation until the biopsy is fully depleted.

Considerations:

1. **FBS concentration:** DMEM supplemented with 20% FBS promotes fibroblast outgrowth, but DMEM with 10% FBS can be used subsequently for routine culture.
2. **Morphological Characteristics:** Fibroblasts typically exhibit elongated, spindle-shaped morphology with thin processes, consistent with their proliferation in fibroblast-specific medium. Unlike keratinocytes, fibroblasts do not require additional supplements or growth factors due to their higher mitotic activity.
3. **Characterization Methods:** Cell characterization of the replication time can be performed using a trypan-blue exclusion assay with a BioRad TC20 Automated Cell Counter (Figure 2). For more robust characterization, immunofluorescence and flow cytometry are recommended (Table 1, Figures 3 and 4).

5.1 **Cellular Propagation:**

- Once fibroblasts cover approximately 30–40% of the well, aspirate the medium and wash the well twice with 1X PBS (Phosphate-buffered saline) to remove the residual medium.
- Aspirate the PBS and add 1 mL of pre-warmed 0.25% Trypsin/EDTA solution to the culture. Incubate for 1 to 2 minutes, avoiding over-trypsinization to prevent cell death. Under an inverted microscope, confirm cell detachment.
- Gently, detach the cells surrounding the tissue explant (TE) by pipetting the trypsin solution, taking care to ensure the TE remains attached to the well.
- Add 1 mL of fibroblast medium to the well to collect the detached cells and transfer the resulting 2 mL of cell suspension into a 25-cm² flask.

- Rinse the well with an additional 3 mL volume of **FBM** to recover any remaining cells and transfer this suspension into the same flask, bringing the final volume to 5 mL.
- Label the flask appropriately and place it in a 37ºC, 5% CO_2 incubator for further culture.

6 **STEP 4: Cryogenic preservation of derived fibroblasts** 20m

Considerations:

To thaw the cell line: Add 20 mL of tempered fibroblast medium to a 75-cm2 flask. Retrieve a vial of frozen fibroblasts from the liquid nitrogen and pipette the cell suspension. Allow the cells to adhere for 3-4 hours and replace the medium to remove residual DMSO.

6.1 **Procedure:**

- Aspirate the medium from a 90-100% confluent 75-cm2 flask and wash the cells twice with PBS.
- Remove the PBS and add 1 mL of pre-warmed trypsin/EDTA solution. Incubate for 1 to 2 min.
- Count the cells with a trypan-blue exclusion assay in a hemocytometer or cell counter.
- Transfer the cell suspension to a 15-ml conical tube and centrifuge at 450 g for 5 min.
- Discard the medium and resuspend the cell pellet in freezing medium (1:1 DMEM low glucose, FBS with 10% DMSO). Adjust the volume to ensure a cell concentration of 2-5·106 cells/mL per cryovial.
- Aliquot 1ml into each cryovial.
- Place the vials into a cryogenic freezing container. The next day, store in liquid nitrogen.

7

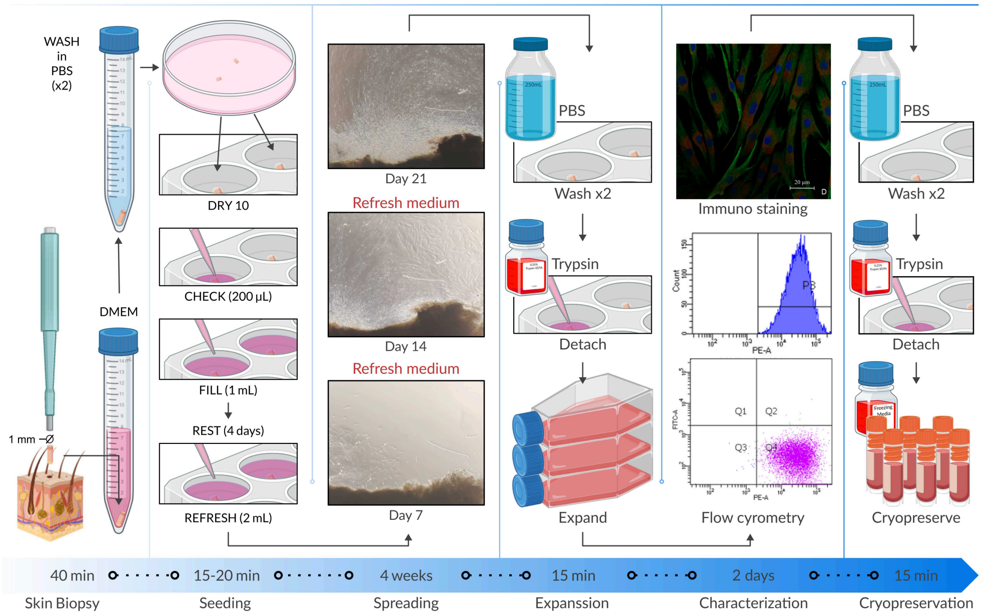

Figure 1. Summary of the protocol, including the four main steps: 1) biopsy extraction, 2) seeding and spreading, 3) expansion, and 4) cryopreservation; followed by the characterization of Primary Fibroblast Cultures.

Characterization of Primary Fibroblast Cultures

8 **Growth Curves**

Given that we have reduced the size of the explant to 1 mm, it is critical to evaluate the viability of the cells over time and assess their proliferation rate to ensure their suitability for molecular biology assays. To address these objectives, we characterized the culture by studying its growth curve. Using a 24-well culture plate, 2.5×10^4 cells were seeded per well and cultured in DMEM supplemented with 20% FBS. Over 11 days, cells from two wells were stained with Trypan blue solution and counted daily using a Bio-Rad TC20™ Automated Cell Counter. The cells demonstrated robust growth, with an approximate doubling time of 24 hours during the exponential phase, before ceasing division upon reaching a confluent state, consistent with the natural growth plateau observed in fibroblast cultures. Importantly, cell viability remained high throughout the experiment, suggesting the cells maintained functionality without immediate signs of senescence, enabling their use for downstream applications within their passage limits.

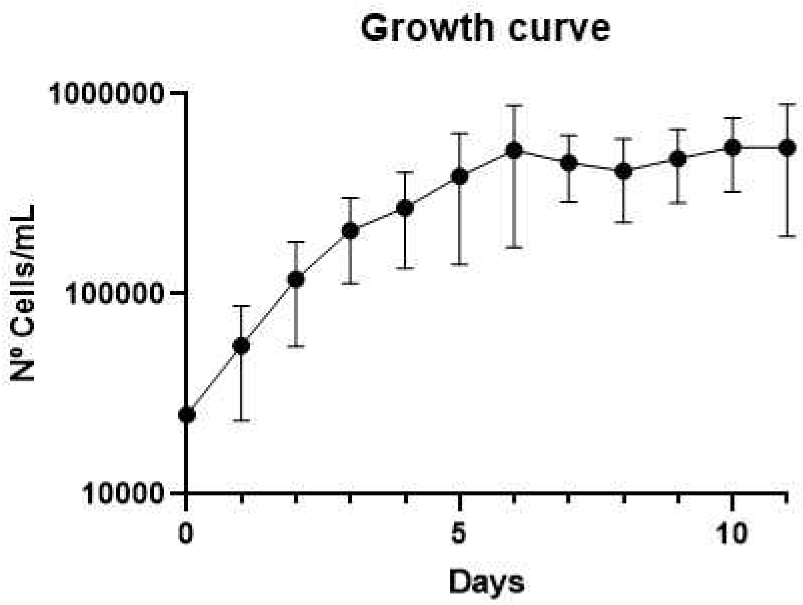

Figure 2. Fibroblast growth curve conducted with five biological replicates. Second-passage human fibroblast cultures were plated in 24-well culture plates and maintained in complete DMEM. Daily, two wells were counted per individual. The mean cell growth and standard error across all individuals are shown. During the initial three days, the cell number doubled approximately every 24 hours. Subsequently, cell numbers stabilized, plateauing at approximately 2–3 × 10^5 cells per well.

9 **Immunofluorescence**

The identity of HDF was assessed by immunofluorescence, using vimentin and human fibroblast surface protein as positive markers and EpCAM/TROP as a negative marker (Figure 3). Fibroblasts were seeded in 12-mm poly-D-lysine coated coverslips, fixed with paraformaldehyde 4% for 15 minutes and permeabilized with 0.1% Triton X-100 for 10 minutes. Following permeabilization, cells were blocked with 5% bovine serum albumin (BSA) for 1 hour and incubated with primary antibodies overnight at 4ºC. After washing, secondary antibodies were applied for 2 hours and nuclei were counterstained with DAPI. Coverslips were mounted using Vectashield, and images were acquired using a ZEISS LSM 900 confocal microscope. Phalloidin staining revealed the characteristic spindle-shaped morphology of HDF. Images confirmed that cells were positive for fibroblast-specific markers, with a high signal from vimentin and human fibroblast surface protein. Moreover, cells were negative for EpCAM/TROP, confirming the absence of epithelial contamination and validating the fibroblast identity and purity of the culture.

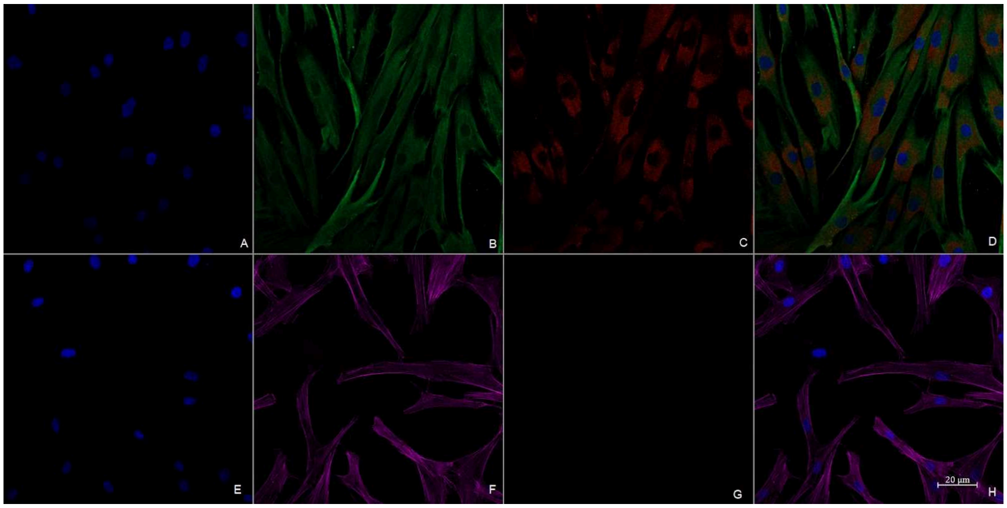

Figure 3. Characterization of HDF derived from skin biopsy by immunofluorescence. Nuclei were counterstained by DAPI (A, E). Fibroblast-specific markers: anti-vimentin (green) (B) and anti-human fibroblast surface protein (red) (C). Merged images A-C (D). F-actin detected with Phalloidin-iFluorTM 647 conjugate (purple) (F). Fibroblast negative marker EpCAM/TROP. (G) Merged images E-G (H). Images were acquired with 20x magnification.

10 **Flow cytometry**

To ensure the purity of the fibroblast primary culture, we conducted flow cytometry analysis, acknowledging that the initial skin biopsy may contain other cell types, including keratinocytes, melanocytes, and immune cells such as lymphocytes or macrophages. We tracked the evolution of cellular subpopulations from the tissue explant stage through successive passages, achieving >99% fibroblast purity by passage 3. At the biopsy stage, cells were passaged upon reaching 70–80% confluence as described previously, with 1×10^6 cells reserved for flow cytometry analysis using a BD FACSCelesta™ SORP cytometer. Monoclonal antibodies S100A4-PE and CD49f-FITC were employed as positive and negative fibroblast markers, respectively. Analysis revealed that the initial cell population was predominantly made up of fibroblasts, with ~88% of cells positive for S100A4. Over successive passages, fibroblast purity progressively increased, reaching >99% by passage 3 (Table 1, Figure 4). These findings confirmed that the culture conditions effectively favored fibroblast enrichment while minimizing contamination from other cell types.

	A	B	C	D	E	F
	**Cellular Population**	CN (%)	Biopsy (%)	P1 (%)	P2 (%)	P3 (%)

	A	B	C	D	E	F
	**S100A+**	0	87.76 ± 14.07	91.52 ± 4.96	99.5 ± 0.23	97.84 ± 1.25

Table 1. Percentages (± SD) of S100A4+ cells from biopsy, passage 1 (P1), passage 2 (P2), and passage 3 (P3) (n=5). CN= negative control.

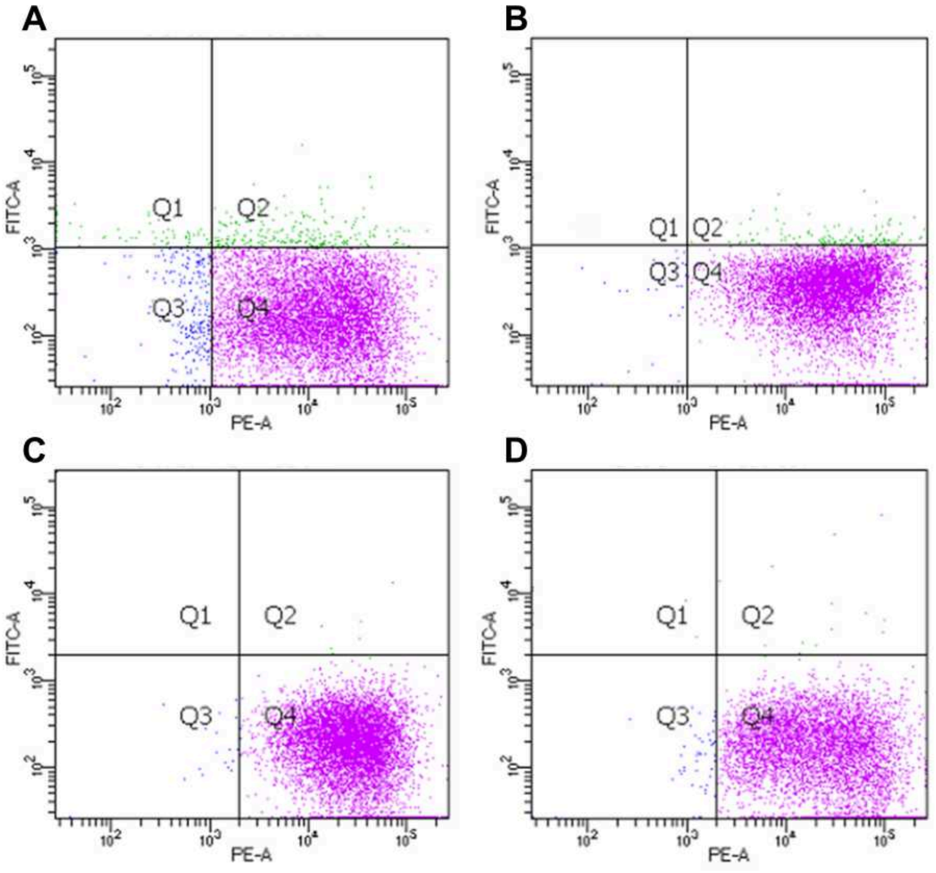

Figure 4. Cellular characterization of cell populations phenotypes obtained from biopsies and passages 1, 2 and 3. (A-D). Representative dot plots (n=5) showing the average percentage of cells labeled with CD49f (FITC-A) and S100A4 (PE-A). Fluorescence thresholds vary across dot plots as they correspond to different individuals, with each threshold determined based on its respective negative control.

Discussion

11 In clinical practice, patient-derived cells enable straightforward analyses, such as protein or transcript quantification. For instance, peripheral blood mononuclear cells can be isolated via ordinary blood sample collection and ficoll separation. However, understanding pathophysiological conditions often requires more comprehensive studies, including cellular response to stimuli, regulatory mechanisms, and the effects of therapeutic agents. Such research requires cultivable cells, which constitute the cornerstone for executing complex molecular biology techniques.

While immortalized cell lines offer convenience, they present significant limitations, including rapid proliferation, accumulation of mutations, and the absence of patient-specific genetic profiles. Moreover, they often require transfection with vectors carrying the mutated protein, leading to gene expression and regulation that may not accurately reflect natural conditions. Therefore, primary cell cultures derived from patient samples represent the gold standard for personalized and accurate characterization. However, the invasive nature for the acquisition of primary cells, particularly in pediatric populations, raises ethical concerns, especially in cases where the immediate benefit to the patient is unclear, such as in rare or mild genetic conditions.

The collection of skin from 1-mm biopsies offers a minimally invasive alternative, providing a time window and cell yield comparable to larger 4-mm biopsies. This approach allows sufficient cell quantities for experimentation or cryogenic preservation within approximately one month. Due to its reduced invasiveness, this method can be performed during routine follow-up visits by trained physicians at the point of care, significantly reducing patient burden. Moreover, the associated protocol is straightforward, cost-effective, and feasible in any facility with basic cell culture capabilities, promoting accessibility and scalability for biomedical studies.

Although existing skin biopsy protocols primarily aim at pathological diagnoses [8,14], the proposed refined method is tailored for pediatric biomedical research, particularly for rare diseases. It facilitates the development of much-needed biobanks [7], enabling functional studies and therapeutic exploration with minimal patient discomfort. This innovative protocol supports diagnostic advancements and personalized medicine, particularly in pediatric care, addressing critical gaps in rare disease research with efficiency and ethical sensitivity.

Protocol references

1. Lunke S, Bouffler SE, Patel CV, Sandaradura SA, Wilson M, Pinner J, et al. Integrated multi-omics for rapid rare disease diagnosis on a national scale. Nat Med. 2023;29: 1681–1691.
2. Faye F, Crocione C, Anido de Peña R, Bellagambi S, Escati Peñaloza L, Hunter A, et al. Time to diagnosis and determinants of diagnostic delays of people living with a rare disease: results of a Rare Barometer retrospective patient survey. Eur J Hum Genet. 2024;32: 1116–1126.
3. Monaco L, Zanello G, Baynam G, Jonker AH, Julkowska D, Hartman AL, et al. Research on rare diseases: ten years of progress and challenges at IRDiRC. Nat Rev Drug Discov. 2022;21: 319–320.
4. Wright CF, FitzPatrick DR, Firth HV. Paediatric genomics: diagnosing rare disease in children. Nat Rev Genet. 2018;19: 325.
5. Landrum MJ, Lee JM, Benson M, Brown GR, Chao C, Chitipiralla S, et al. ClinVar: improving access to variant interpretations and supporting evidence. Nucleic Acids Res. 2018;46: D1062–D1067.
6. Richards S, Aziz N, Bale S, Bick D, Das S, Gastier-Foster J, et al. Standards and guidelines for the interpretation of sequence variants: a joint consensus recommendation of the American College of Medical Genetics and Genomics and the Association for Molecular Pathology. Genet Med. 2015;17: 405–424.
7. Li Y, Polak U, Clark AD, Bhalla AD, Chen Y-Y, Li J, et al. Establishment and Maintenance of Primary Fibroblast Repositories for Rare Diseases-Friedreich's Ataxia Example. Biopreserv Biobank. 2016;14: 324–329.
8. Villegas J, McPhaul M. Establishment and culture of human skin fibroblasts. Curr Protoc Mol Biol. 2005;Chapter 28: Unit 28.3.
9. Kim SH, Kim JH, Lee SJ, Jung MS, Jeong DH, Lee KH. Minimally invasive skin sampling and transcriptome analysis using microneedles for skin type biomarker research. Skin Res Technol. 2022;28: 322–335.
10. Wang CY, Maibach HI. Why minimally invasive skin sampling techniques? A bright scientific future. Cutan Ocul Toxicol. 2011;30: 1–6.
11. Takashima A. Establishment of fibroblast cultures. Curr Protoc Cell Biol. 2001;Chapter 2: Unit 2.1.
12. Iannello G, Patel A, Sirabella D, Diaz AG, Hoover BN, Sarmah H, et al. Simple, Fast, and Efficient Method for Derivation of Dermal Fibroblasts From Skin Biopsies. Curr Protoc. 2023;3: e714.
13. Hayflick L. THE LIMITED IN VITRO LIFETIME OF HUMAN DIPLOID CELL STRAINS. Exp Cell Res. 1965;37: 614–636.
14. Vangipuram M, Ting D, Kim S, Diaz R, Schüle B. Skin punch biopsy explant culture for derivation of primary human fibroblasts. J Vis Exp. 2013; e3779.
