# Supplementary material for: A minimally invasive lab protocol for fibroblasts isolation from 1-mm skin punch biopsies in pediatric patients
Source: PLoS One. 2026 Jun 22;21(6):e0350828. doi: 10.1371/journal.pone.0350828 (PMC13286191; doi:10.1371/journal.pone.0350828)
Supplement: Supplementary file 4 [file pone.0350828.s002.odt]

# Reagents and Materials

| Reagents and Materials | Source | Reference |
| --- | --- | --- |
| Cell culture |
| DMEM low glucose | PanBiotech | P04-01550 |
| Fetal Bovine Serum (FBS) | Corning | 35-079-CV |
| Penicillin-Streptomycin | PanBiotech | P06-07100 |
| 0.25% Trypsin/EDTA | PanBiotech | P10-019500 |
| Dimethylsulfoxide (DMSO) for cell culture | PanReac AppliChem | A3672 |
| 15 mL conical falcon | Deltalab | 429942 |
| 22-cm2 Petri dish | Soria Genlab | P101 |
| 6-well culture plate | Sarstedt | 83.3920 |
| 24-well culture plate | Sarstedt | 83.3922 |
| Sterile 12 mm glass coverslip | Labolan | 20012 |
| Poly-D-lysine | Sigma-Aldrich | P1024 |
| Immunofluorescence |
| Monoclonal Anti-Human Fibroblast Surface Protein (1:400) | Sigma | F4771 |
| Vimentin (1:500) | NovusBio | NB300-223 |
| EpCAM/TROP-16 | Biotechne (R&D System) | AF960 |
| Goat anti-Mouse IgG (H+L) Cross Adsorbed Secondary Antibody, Alexa Fluor TM 568 (1:1000) | Invitrogen | A-11004 |
| Goat anti-Chicken IgY (H+L) Cross Adsorbed Secondary Antibody, Alexa Fluor TM 488 (1:1000) | Invitrogen | A32931 |
| Donkey Anti-Goat IgG H&L (Alexa Fluor 750) (1:1000) | Abcam | ab175744 |
| Phalloidin-iFluorTM 647 conjugate | Cayman Chemical | 20555 |
| DAPI | Santa Cruz | sc-3598 |
| Flow cytometry |
| CD49f Antibody, Anti-human/mouse, FITC | Miltenyi Biotec | 130-097-245 |
| PE anti-human, S100A4 Antibody | BioLegend | NJ-4F3-D1 |
| 5 mL Polystyrene Round-Bottom Tube | Corning | 352052 |
| Skin Biopsy |
| Biopsy Punch 1 mm | Kai Medical | BP-10F |
| EMLA (Anesthetic cream) | Aspen | 679290 |
| Prontosan (Antiseptic) | Braun | 400403 |
| Adhesive bandage | IHT | 0037107 |
| Sterile gauze | Texpol | 1186.01 |
| Sterile tweezers | Medical Waitch | 166 |
| Equipment |
| Laminar Flow Hood | Holten LaminAir | Model 1.2 |
| Water bath, 37ºC | Trade Raypa |  |
| Incubator 37ºC, 5% CO2 | Thermo Scientific | HeraCell 150 |
| Centrifuge | Eppendorf | 5810R |
| Confocal Microscope | Zeiss | Axio Observer |
| Inverted Phase-Contrast Microscope | Olympus | CKX41 |
| Cytometer, FACS CELESTA SORP | Becton Dickinson |  |
